# Vigour testing for the rice seed with computer vision-based techniques

**DOI:** 10.3389/fpls.2023.1194701

**Published:** 2023-09-18

**Authors:** Juxiang Qiao, Yun Liao, Changsheng Yin, Xiaohong Yang, Hoàng Minh Tú, Wei Wang, Yanfang Liu

**Affiliations:** ^1^ Quality Standard and Testing Technology Research Institute, Yunnan Academy of Agricultural Sciences, Kunming, China; ^2^ Software School, Yunnan University, Kunming, China; ^3^ Seed Management Station of Yunnan Province, Kunming, China; ^4^ National Center for Testing and Testing of Plant Seeds and Products, Hanoi, Vietnam

**Keywords:** rice seed vigour, multispectural imaging, MsiFormer, TTC staining, nCDA

## Abstract

Rice is the staple food for approximately half of the world’s population. Seed vigour has a crucial impact on the yield, which can be evaluated by germination rate, vigor index and *etc*. Existing seed vigour testing methods heavily rely on manual inspections that are destructive, time-consuming, and labor-intensive. To address the drawbacks of existing rice seed vigour testing, we proposed a multispectral image-based non-destructive seed germination testing approach. Specifically, we collected multispectral data in 19 wavebands for six rice varieties. Furthermore, we designed an end-to-end pipeline, denoted as MsiFormer (MisFormer cod3e will be available at https://github.com/LiaoYun0x0/MisFormer) by integrating a Yolo-based object detector (trained Yolo v5) and a vision transformer-based vigour testing model, which effectively improved the automation and efficiency of existing techniques. In order to objectively evaluate the performance of the proposed method in this paper, we conduct a comparison between MisFormer and other 3 deep learning methods. The results showed that, MisFormer performed much better with the accuracy of 94.17%, which was 2.5%-18.34% higher than the other 3 deep learning methods. Besides MsiFormer, possibilities of CIELab mediated image analysis of TTC (tetrazolium chloride) staining in rice seed viability and nCDA (normalized canonical discriminant analysis) in rice seed vigour were also discussed, where CIELab L^*^ of TTC staining were negatively correlated with vigor index and germination rate, with Pearson’s correlation coefficient of -0.9874, -0.9802 respectively, and CIELab A^*^ of TTC staining were and positively correlated with vigor index and germination rate, with Pearson’s correlation coefficient of 0.9624, 0.9544 respectively, and CIELab A^*^ of nCDA had Pearson’s correlation coefficient of -0.8866 and -0.9340 with vigor index and germination rate, respectively. Besides testing methods, vigour results within and among variety(ies) showed that, there were great variations among the 6 rice varieties, and mean coefficient of variation (CV) of vigor index of individual seed within a variety reached 64.87%, revealing the high risk of conventional methods in random sampling. Vigour variations had close relationship with wavelengths of 780 nm-970 nm, indicating their value in future research.

## 1 Introduction

Rice (*Oryza sativa* L.), the staple food consumed by about half of the world’s population, is of great importance due to its various nutrients and caloric contribution (Chen et al., 2022), as well as its model plant role in monocot genetics (Flavell, 2009). In China, rice yield account for 40% of total grain output, where planting area of hybrid rice has exceeded 50% of rice planting (Luo, 2021). Seed vigour is the sum of those properties that determine the activity and performance of seed lots of acceptable germination in a wide range of environments. Growing concerns about improving rice seed vigour has been observed over the past few decades since rice production becomes mechanized, with direct sowing replacing manual transplanting (Liang et al., 2022). Furthermore, seed vigour should receive the utmost attention in order to address issues with climate change and land depletion because it can ensure rapid and uniform germination and subsequent seedling development to survive under the harsh environments, and increase productivity of arable lands (Havé et al., 2017; Kishor et al., 2021). Seed vigour deserves more priority than investments on fertilizers, pesticides and other inputs since it is the first step in crop surviving and has impact on yield and field management. However, seed vigour is less mentioned compared with disease control, cultivation management, *etc*.

Seed vigour is a comprehensive concept, and it has tight connection with the potential of seed germination and field performance (Sun et al., 2007; Baalbaki, 2009), and is generally explained via germination rate, vigor index and *etc.* Germination test and seedling growth test are still used as the official method for evaluating seed vigour in most countries which are based on random sampling (small sampling representing batches or bulks of seeds) (Agelet et al., 2012). Seed viability (germination capacity) was defined as the percentage of germinated seeds (Men et al., 2017), and generally tested by tetrazolium chloride (TTC) staining. Generally, the seed vigour high, its viability is also high. TTC staining has been proven its efficiency in assessment of seed viability of castor bean seeds (Gaspar-Oliveira et al., 2010; Diantina et al., 2022). However, the quantitative correlation between intensity of TTC staining and seed viability is unknown.

Phenomics is increasingly applied in seed industry (Fan et al., 2023). Multispectral imaging (MSI) is an emerging technology that integrates imaging and spectroscopy to obtain both spatial and spectral information of the target objects simultaneously (Mastrangelo et al., 2019; França-Silva et al., 2020). MSI can provide information of chemical composition, and phenotypic features (texture, color, shape, size) based on reflected and absorbed light information of interior and surface materials of seeds (Xia et al., 2019; ElMasry et al., 2019a; Mortensen et al., 2021). Since sample pretreatments are not a necessity, MSIs provide the great potential for nondestructive and straightforward measurement in a rapid and robust manner. MSI was initially used to screen transgenic rice seeds (Liu et al., 2014) and discriminate rice varieties (Liu et al., 2016). Currently, this technology has been increasingly promising, e.g. detection of physiological and physical properties of seeds of peanut (Oliveira et al., 2022), soybean (Silva et al., 2021), tomato and carrot (Galletti et al., 2020), identifying of hard and soft seeds of six legume species (Hu et al., 2020), discrimination of different aged and germinated seeds of cowpea (ElMasry et al., 2019b), assessment of seed viability of castor beans (Olesen et al., 2015). Compared with other cash crops, seed vigour is less discussed in rice.

MSI technology in seed testing usually involves multiple steps: sample preparation, device calibration, image and data acquisition, ROI (regions of interest) segmentation, feature extraction, analysis and modelling (Mortensen et al., 2021). For practice purpose, seeds are typically photographed in bulk instead of single seed. Individual seed testing is in great favor of accuracy of the whole sample and removing of unqualified seeds (Li et al., 2018; ElMasry et al., 2019a). Traditional ROI segmentation is done by hand or with the aid of cluster algorithms, which is time-consuming, or dependent on threshold setting. Effective ROI segmentations are few reported (Li et al., 2018; ElMasry et al., 2019a). In the stage of modelling, linear discriminant analysis (LDA), artificial neural network (ANN), support vector machine (SVM) and least squares discriminant analysis (PLS-DA) are usually employed (Xia et al., 2019; ElMasry et al., 2019a; Mortensen et al., 2021). Along with the development of deep learning, more sophisticated algorithms are expected to enhance prediction accuracy of the models.

In our study, research was carried out to reveal the possibility MSI technology in rice seed vigour testing. Six rice varieties were used and treated with 0-, 5-, 10- and 20-day accelerated ageing to generate vigour gradient artificially. Multispectral images and corresponding data of rice seeds were acquired taking advantage of the 19 wavelengths of Videometer Lab4™ instrument. Dataset was constructed based on ROI segmentation, data augmentation and data merging. Taking the data of germination rate of the 6 rice varieties as training and testing samples, a new deep learning network, *i.e.* prediction model was developed, which was combining residual convolutional structure and Transformer. Performance of our prediction model was evaluated compared with some classical deep learning methods. Finally, MsiFormer was proposed in our study for the purpose of germination detecting of rice seeds. Besides MsiFormer, this paper also revealed the possibility of CIELab-mediated analysis of TTC staining images in viability testing and nCDA (normalized canonical discriminant analysis) images in vigour testing of rice seeds. Vigour variations and their correlations with reflectance features and morphological features were investigated as well.

## 2 Materials and methods

### 2.1 Materials

Totally 6 varieties were used in our study, which were from ‘Rice Diversity Panel’ (RDP) established by Genetic stocks Oryza (GSOR) of Ministry of Agriculture of the U.S.A (McCouch et al., 2016) and were propagated in Yunnan in 2020. Among the 6 varieties, 3 varieties belong to *japonica* subgroup, 1 variety belongs to *indica* and 1 *aus* rice (**Table 1**).

**Table 1:** Information of the 6 rice varieties.

Code	Variety Name	Subpopulation	Country of origin
CY-7	JHONA 26	*aus*	Pakistan
CY-80	KALIBORO::IRGC77201-1	NA	Bangladesh
CY-150	BOTRA FOTSY::IRGC77840-1	*tropical-japonica*	Madagascar
CY-203	WU KE NUO::IRGC59990-1	*temperate-japonica*	China
CY-229	JOALBHANGA 499::IRGC6560-1	*indica*	Bangladesh
CY-256	KYEEMA::GERVEX 1656-C1	*tropical-japonica*	Australia

NA: it was unclear which subgroup the variety belongs to.

### 2.2 Accelerated ageing

Accelerated ageing was adopted to generate vigour gradients artificially. Seeds of each variety were placed smoothly in one layer in sealing bags and incubated in 42°C water bath for 5 days, 10 days and 20 days. After accelerated ageing, seeds were incubated at 25°C for 1 week before image collection. (Baek et al., 2019)

### 2.3 Multispectral image analysis on seed vigour

#### 2.3.1 Pipeline for fully automatic seed germination prediction

**Figure 1:**
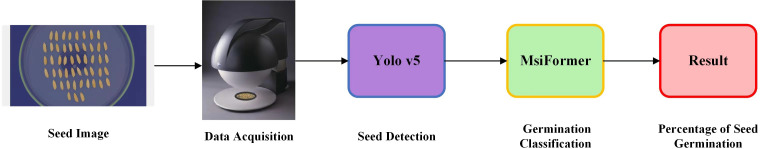
The pipeline for fully automatic seed germination prediction.

In order to efficiently perform end-to-end non-destructive seed germination detection, a pipeline as shown in **Figure 1** was proposed. Firstly, the seed images were collected through Videometer Lab4™ for data acquisition, which can obtained a large number of multispectral images. Secondly, the object detection of image data was performed by Yolo v5, and the ROI segmentation of individual seed was implemented. Then, the extracted multispectral image of each seed was input into the designed MsiFormer for predicting germination. Finally, the final germination prediction results of individual seeds were obtained by MsiFormer.

#### 2.3.2 Image and data acquisition

Raw multispectral images were captured by a Videometer Lab4™ instrument (Videometer A/S, Herlev, Denmark), which consists of 19 wavelengths (365, 405, 430, 450, 470, 490, 515, 540, 570, 590, 630, 645, 660, 690, 780, 850, 880, 890 and 970 nm). Videometer Lab4™ consists of a 5 mega pixel CCD camera, mounted inside the top of the integrating sphere, coated with highly white and diffusing paint and illumination by narrowband high-power LED placed at the rim, and thereby ensures uniform diffuse lighting, and minimizes shadows or specular reflection. Before capturing images, calibration was conducted using 3 discs, *i.e.* the bright disc for reflection calibration, the dark disc for background calibration and the dotted disc for aligning calibration of geometric pixel position. Light configuration was also calibrated to optimize the intensity at each wavelength. In ‘light setup’, 100% reflectance was selected in our study.

For each acquisition, 50 seeds were placed evenly in a 9-cm glass petri dish, which were then placed at the bottom of the integrating sphere. Then 20 high resolution multispectral images of 2192 × 2192 pixels were captured (19 spectral images with different wavelengths and 1 image in RGB form) within 5-10 seconds. Six varieties were applied for multispectral image analysis, and for each variety, 200 seeds were used.

Besides images, corresponding data were also obtained, including reflectance features and morphological features, *e.g.* area, length, width, ratio width/length, compactness circle, compactness ellipse, betashape a, betashape b, vertical skewness, CIELab L*, CIELab A*, CIELab B*, saturation, hue, vertical orientation.

#### 2.3.3 ROI segmentation

In the proposed pipeline, Yolo v5 was used to segment the pictures of each individual seed. Yolo v5 is a very effective deep learning-based object detection algorithm. It can automatically identify the categories and locations of different objects in the image, and label the bounding box of each object accurately.

In order to train Yolo v5, 240 rice seeds were collected from each of the six varieties, namely CY-7, CY-80, CY-150, CY-203, CY-229, and CY-256. After accelerated aging processing, multispectral images were taken respectively.

Then the above images were manually annotated with the bounding boxes of the seeds. We labeled 6 RGB images without aging. Each image contained 50 seeds, so we labeled 300 samples in total. Finally, transfer learning was performed based on Yolo v5. Afterwards, the IOU loss coefficient was set to be 0.05 and the CLS loss coefficient to be 0.5. After 5 cycles of training, the model could accurately detect the bounding box of the seed in the petri dish.

#### 2.3.4 MsiFormer

##### 2.3.4.1 Dataset for germination prediction

Based on the trained Yolo v5 model, 24,000 seed images (6 varieties, 200 seeds for each variety, and 20 images for each seed) were obtained. Finally, the uniform sampling strategy was used to obtain 100 germinated seed samples and 100 non-germinated seed samples from the samples uniformly. 20000 images (1000 seeds) were selected for training and 4000 images (200 seeds) for testing.

**Figure 2:**
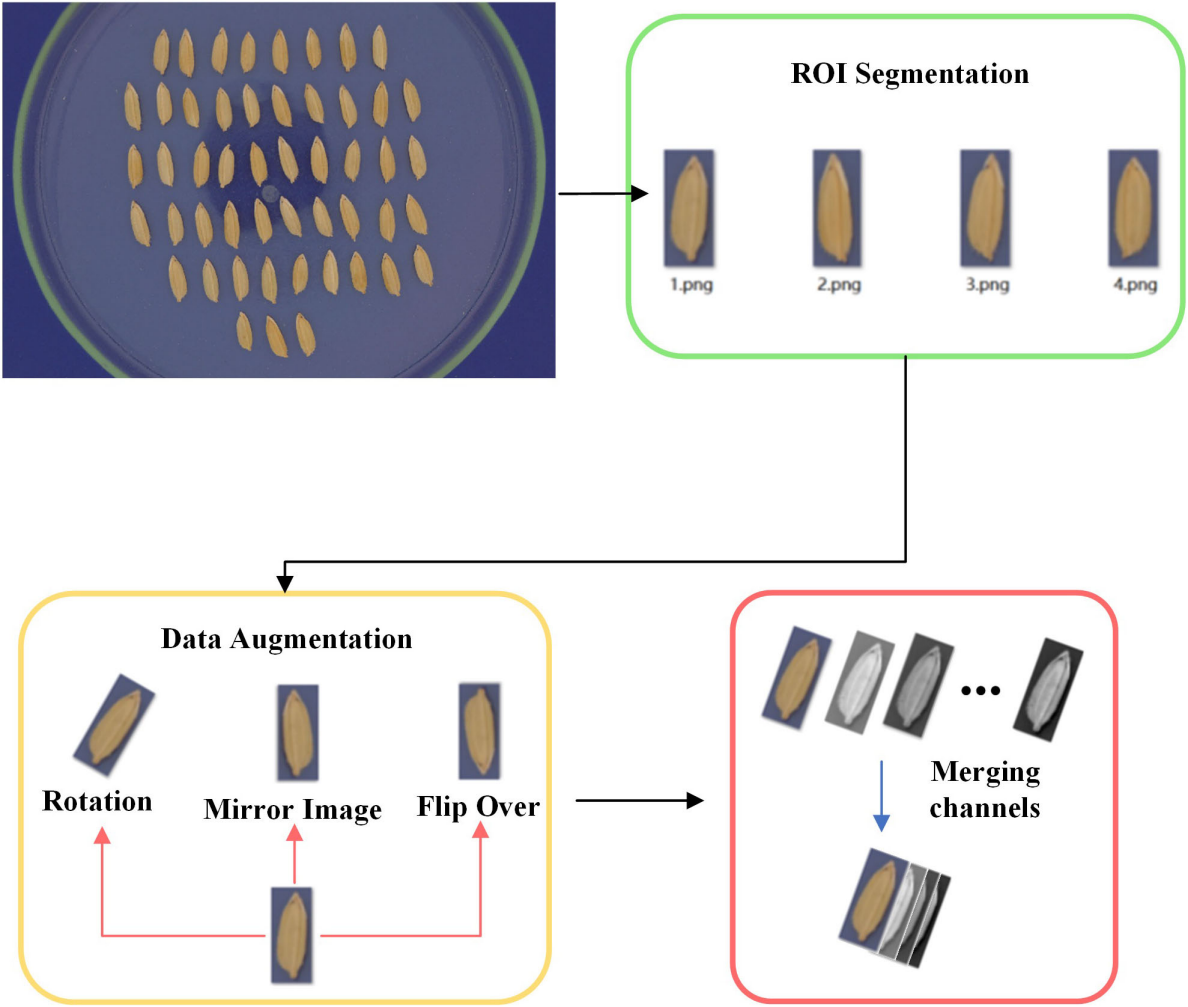
Illustration of the dataset construction.

Data augmentation was applied for dataset construction (**Figure 2**). After ROI segmentation, images of individual seed were rotated, mirrored and flipped, for the purpose of enriching the training data and improving the learning ability of the algorithm for complex data. Finally, images were resized to 256*256, and channels of RGB image and 19 spectral images were merged to generate 22 channels of multi-channel data.

##### 2.3.4.2 Modelling for germination prediction

A new deep learning network (MsiFormer) combining residual convolutional structure and Transformer for germination prediction was proposed in our study.

According to **Table 2**, the network accepted images of size (256, 256, 22) and outputs features of size (8, 8, 2048) from layers 0 to 4 of the designed residual network structure. The global features of images were further learned by the self-attention module of the Transformer, and features of size (1, 1, 2048) were output. The residual block of our designed deep residual network used skip connections, which relieved the problem of gradient disappearance brought by increasing depth in deep neural networks, and improved the accuracy by increasing a comparable depth. Self-attention was used in our vision Transformer module to make each pixel related to other pixels, which greatly enhanced their correlation with seed germination.

**Table 2:** Description on deep convolutional networks stage.

Layer	Layer Description	Output Tensor (h*w*n)
Input	Input image patch	256*256*22
Stage 0 (CNN)	Conv(3*3), stride(2), padding(1) Conv(3*3), stride(2), padding(1)	64*64*64
Stage 1 (CNN)	Conv(3*3), stride(1)	64*64*256
Stage 2 (CNN)	Conv(3*3), stride(2), padding(1)	32*32*512
Stage 3 (CNN)	Conv(3*3), stride(2), padding(1)	16*16*1024
Stage 4 (CNN)	Conv(3*3), stride(2), padding(1)	8*8*2048
Transformer	Self-attention	8*8*2048
Output	Conv(8*8), stride(1)	1*1*2048

"*" means multiplication.

**Figure 3:**
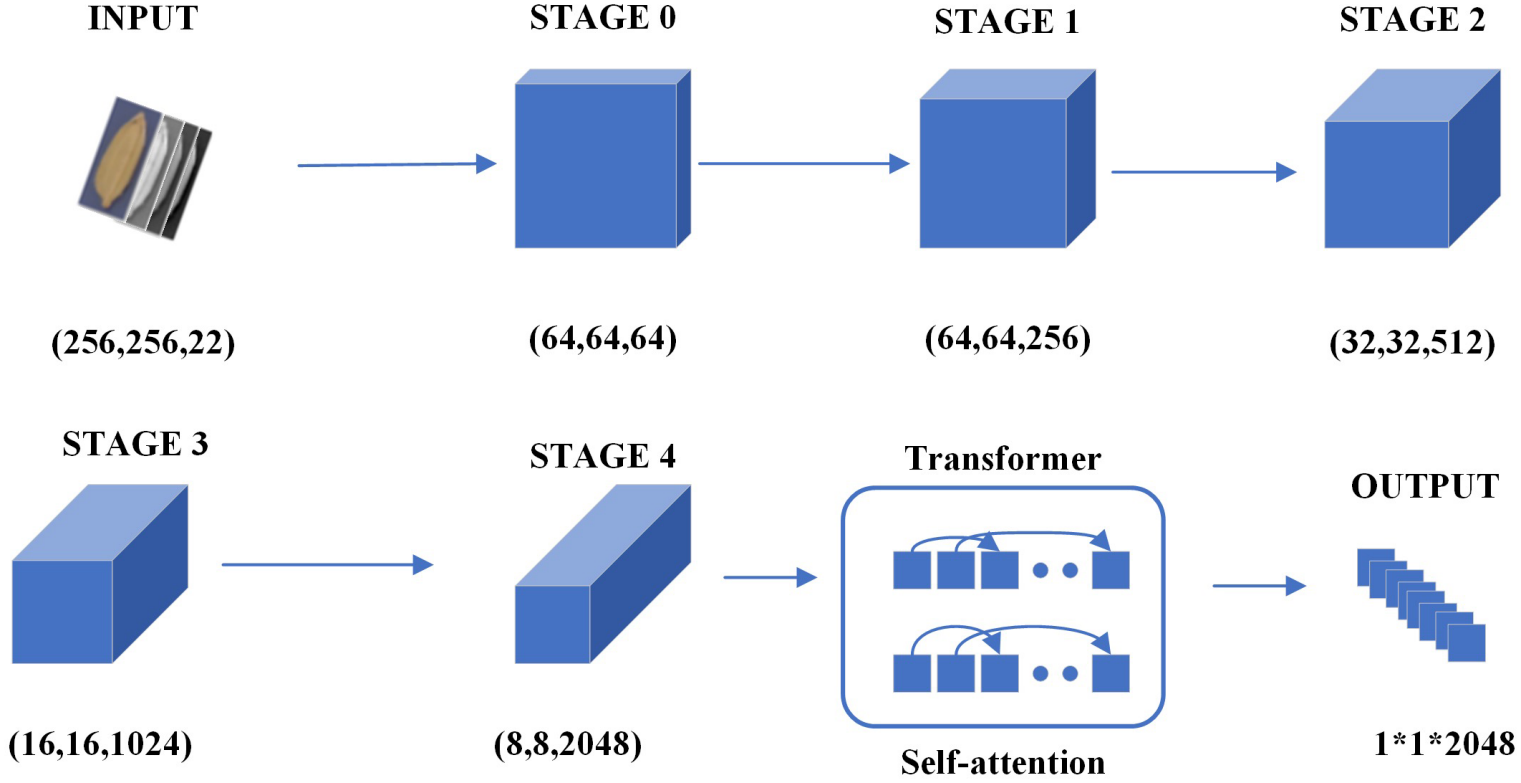
Architecture of MisFromer used in germination prediction. "*" means multiplication.

**Figure 3** briefly showed the architecture of the neural network used for germination prediction purposes. The input to the deep learning model was an image with height of 256, width of 256, and channels of 22, while the output was a feature map with height of 1, width of 1, and channels of 2048 (**Figure 3**). Our deep learning backbone network adopted the idea of combining residual structure and Transformer. The residual structure was initially used to design five large residual convolution layers to perform residual convolution operation on the feature map and learn the feature information of the seed. The skip connection of residual structure was used in each convolutional layer, which greatly reduced the loss of details of features and solved the problem of gradient disappearance and gradient explosion of convolutional networks.

Feature maps with length 8, width 8 and number of channels 2048 were output from the final convolutional layer. A “self-attention mechanism” was used to design a Transformer architecture, which greatly improved the relevance of each feature information in the seed feature map. The designed architecture used five self-attention layers. Unlike convolutional neural networks, Transformers did not change the size and number of channels of the data, but directly extracted features through global attention. Different from the traditional transformer, linear attention (Katharopoulos et al., 2020) was used to replace dot-product attention, where the dot-product attention used an exponential kernel and the linear attention used an alternative kernel function $sim\left(Q,K\right)=\varphi \left(Q\right)\cdot \varphi {\left(K\right)}^{T}$ , where φ(·)=elu(·)+1 . Linear attention can reduce the computational complexity from $O\left(N^{2}\right)$ to O(N) , which greatly improved the operation speed of the model. Afterwards, the size of the output data and the number of channels were still 8*8*2048. With the Transformer structure, the size of the feature map was further reduced and the number of channels was unchanged. The final output was a feature map of (1, 1, 2048).

The feature maps generated by the deep learning model were output to the fully connected layer. All the previously extracted features were integrated and classified by the cross-entropy function. The softmax function was first used to output the probabilities of germinant and non-germinant seeds with values of ‘0’ and ‘1’, respectively. When calculating the final loss of model sprouting, cross-entropy loss function was designed to evaluate the accuracy of softmax to further calculate the logarithmic loss. N was the number of samples; $y_{i}$ presented the label of the ground truth of the sample i , *i.e.* 1 for positive class and 0 for negative class; p denoted the probability that sample i was predicted to be positive.


(1) $$L=\frac{1}{N}\sum {\;}_{i}L_{i}=\frac{1}{N}\sum {\;}_{i}\left[y_{i}\cdot \log\left(p_{i}\right)+\left(1-y_{i}\right)\cdot \text{log}\left(1-p_{i}\right)\right]$$

### 2.4 Biological analysis on seed vigour

#### 2.4.1 Conventional seed vigour testing

Seed vigour test was conducted according to the GB/T 3543.4-1995 (National Standards of P. R. China, 1995) to discriminate the viable seeds from the non-viable seeds. After multispectral imaging, seeds were transferred in sequence to another dish lined with 2 pieces of wet sterilized filter paper, and incubated for 10 days in growth chamber (28°C at day and 25°C at night with a 14-h/10-h light/dark photoperiod). Germinated seeds and corresponding germination time were individually recorded everyday based on the standard of root length ≥ 2 mm. On the 5^th^ day, shoot lengths of each seed were measured, and vigor index and germination rate were calculated according to Song method (Song et al., 2015).

#### 2.4.2 Tetrazolium chloride staining

After accelerate aging, rice seeds (CY-7, CY-80, CY-150, CY-203, CY-229, CY-256) were hulled and soaked in sterile water, and then incubated at 25°C dark condition for 16 h. After water on the seed surface was removed with filter paper, seeds were stained with 1% TTC for 3 h, and washed with PBS buffer (pH 7.0) for 3 times (Olesen et al., 2015; National Standards of P. R. China, 2017; Chen et al., 2021). Seeds were cut in half lengthways, and the embryo staining was observed, where red embryos indicated viable seeds. Staining percentages were calculated based on the stained seeds. There were 3 replicates for each treatment, and 30 seeds for each replicate.

To determine the quantitative correlation between staining intensity of each seed and corresponding seed viability, CY-150 was used. After staining and cutting, the stained embryos were observed and photographed using stereoscope (Leica, M205FC). To determine the staining intensity and staining area, images were analyzed by our software with independent intellectual property rights (Liu, 2022), which can do the quantitative analysis on color via color space conversion from RGB to CIELab.

### 2.5 Statistic analysis

To analyze the correlation between seed vigour and multispectral Imaging, software packages of python and R language (version 4.2.1) was applied, including nCDA, tidyverse, ggplot2, WGCNA, ggsci, *etc*.

To evaluate the performance of our modelling, four types of metrics were applied, including True Positives (TP, prediction is positive and correct), False Positives (FP, prediction is positive and wrong), True Negative (TN, prediction is negative and correct) and False Negative (FN, prediction is negative and wrong). Based on the 4 types of metrics, Accuracy (ACC), Precision, True Positive Rate (TPR), False Positive Rate (FPR), True Negative Rate (TNR), False Negative Rate (FNR) were also used for further evaluation.

## 3 Results

### 3.1 Seed vigour testing based on conventional methods

**Figure 4:**
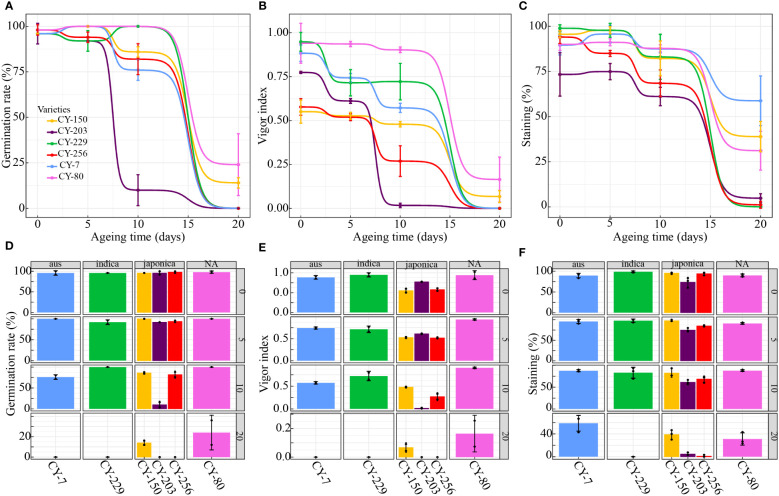
Germination rate **(A, D)**, vigor index **(B, E)** and TTC staining **(C, F)** of 6 rice varieties after treatments of 0-, 5- 10- and 20-day accelerated ageing).

**Figure 4** showed that, seed vigour of the 6 rice varieties were generally dropping, including germination rate, vigor index and TTC staining. There were significantly positive correlations between germination rate and vigor index (Pearson’s correlation coefficient: 0.9101), between germination rate and TTC staining (Pearson’s correlation coefficient: 0.8929) and between vigor index and TTC staining (Pearson’s correlation coefficient: 0.8170). Besides the general dropping trend, there were also variations within and among the 6 varieties. The mean coefficient of variation (CV) of germination rate and vigor index of individual seed of the 6 varieties were 11.18% and 64.87%, respectively, indicating the high risk in seed vigour testing via small sampling representing batches or bulks of seeds.

As for variety, CY-80 seeds kept the highest vigour under 0-, 5- or 10-day accelerated ageing (**Figure 4**); CY-80, CY-7, CY-150, CY-256 and CY-229 dropped their vigour much less after 10-day accelerated ageing than those after 20-day accelerate ageing; CY-203 had obviously dropping vigour after 10-day accelerated ageing compared with those after 20-day accelerated ageing (**Figure 4**). Therefore, the 4 types of accelerated ageing had different impact on the 6 varieties, as well as on individual seed within the variety.

Comparison among the results of germination rate, vigor index and TTC staining showed that, germination rate of the 6 varieties were high (after 0- or 5-day accelerated ageing) and low (after 20-day accelerated ageing) as a whole, with little difference (**Figures 4A, D**), while vigor index of the 6 varieties showed great difference after 0-, 5- and 10-day accelerated ageing, with the most obvious difference after 10-day accelerated ageing (**Figures 4B, E**), indicating vigor index had more potential in distinguishing different viable seeds in early stages, which was in correspondence with the fact that seeds with germination power may lose the capability of growing (Corbineau et al., 2010; Olesen et al., 2015).

It was interesting to note that, CY-203 had low germination rate (10%) and vigor index (0.0164) after 10-day accelerated ageing, but moderately high TTC staining (61.11%), and similar inconformity was also found in vigor index (0) and TTC staining (58.73%) of CY-7 after 20-day accelerated ageing. The reasons may be that, the stained cells had no responsibility to or had lost the capability to germinate or grow despite their ability to respire.

### 3.2 Seed vigour testing based on RGB imaging analysis

**Figure 5:**
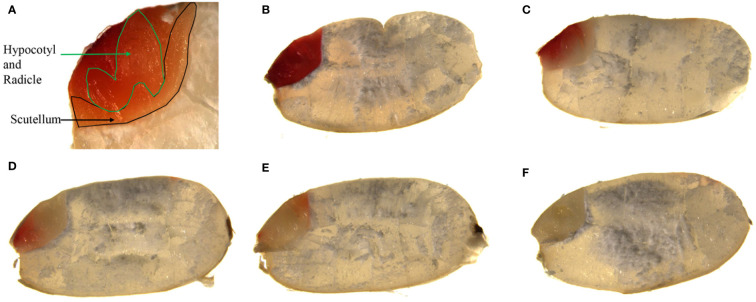
TTC staining of rice variety CY-150 after accelerated ageing. **(A)**: structure diagram of embryo of rice seed after TTC staining; **(B–F)**: indication of intensity and distribution of TTC staining of the whole embryo.

**Figure 6:**
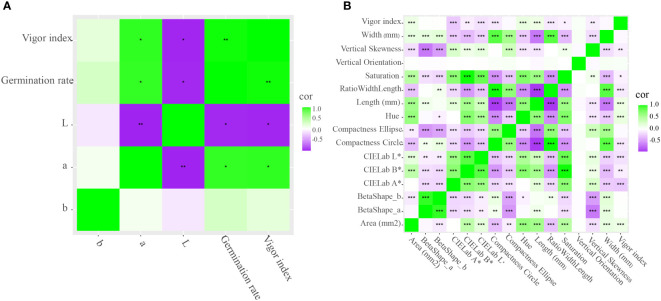
Correlation between vigor index and TTC staining **(A)** as well as morphological features **(B)**. **(A)**: after accelerated ageing and TTC staining, correlation between vigor index of CY-150 and CIELab L^*^, CIELab A^*^, CIELab B^*^ of staining images was concluded; **(B)**: after accelerated ageing, correlation between vigor index of the 6 rice varieties and the 15 morphological features was analyzed. *, **, ***: significant correlation with *p*<0.05, *p*<0.01, *p*<0.001, respectively.

To further explore the correlation between vigor index (or germination rate) and TTC staining, RGB image analysis of staining of seed embryo was conducted. **Figure 5** illustrated that, the intensity of TTC staining became weak gradually (**Figure 5**). CIELab values of TTC staining had significant correlation with vigor index, and germination rate as well, where CIELab L^*^ was negatively correlated with vigor index and germination rate, with Pearson’s correlation coefficient of -0.9874, -0.9802 respectively, and CIELab A^*^ was positively correlated with vigor index and germination rate, with Pearson’s correlation coefficient of 0.9624, 0.9544 respectively, (**Figure 6A**), indicating the potential of CIELab analysis of TTC staining images in the testing of seed viability. Furthermore, according to our results, TTC staining was always weakening from inside to outside, rather than weakening as a whole, indicating cell ageing of scutellum may contribute little to viability loss, while ageing of hypocotyl and radicle may play a major role. Since staining time in our study was ensured, above weakening features in intensity and distribution revealed the cell ageing of individual seed was unsynchronous.

Besides TTC staining, correlation between vigor index of individual seed and morphological features was also analyzed. **Figure 6B** indicated that, CIELab L^*^ and CIELab A^*^ had negative correlation with vigor index, with Pearson’s correlation coefficient of -0.1513 and -0.2756, respectively, at the level of P<0.001. Since the correlation coefficient were not very high and besides, instead of natural aging, heating (42°C) was used for accelerated ageing purpose in our study, whether heating made the hull color changed was unknown. Therefore, above correlation between external color change and vigour difference inside need to be further explored.

### 3.3 Seed vigour testing based on multispectral imaging analysis

**Figure 7:**
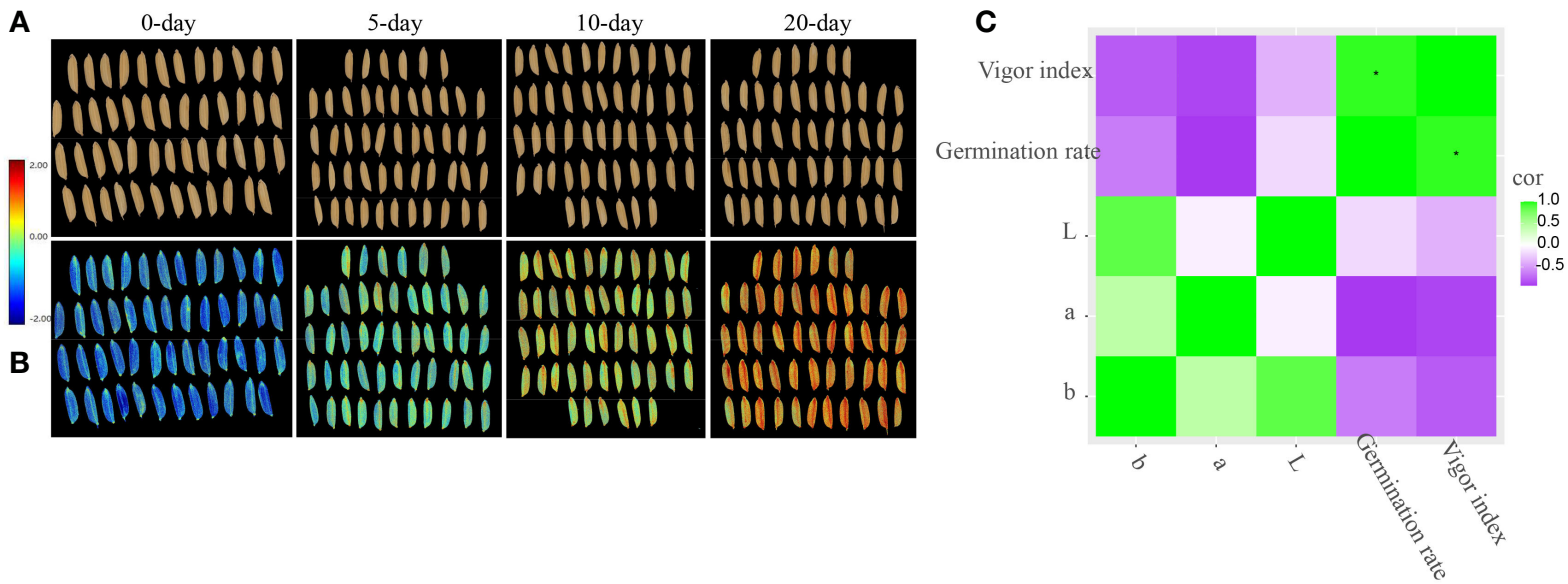
Overview of seeds of CY-256 after 0-, 5- 10- and 20-day accelerated ageing. **(A)**: RGB image; **(B)**: transformed image by nCDA (normalized canonical discriminant analysis); **(C)**: correlation between CIELab L^*^, CIELab A^*^, CIELab B^*^ of nCDA images and vigor index, germination rate.

According to **Figure 7**, nCDA was able to distinguish the 4 groups of seeds with different ageing duration into blue, green, yellow and red as a whole, which was in general correspondence with their vigour results. The ability of nCDA in global assessment of seed vigour was also reported in researches of peanut, castor, et al. (Olesen et al., 2015; Oliveira et al., 2022).

Besides global assessment, CIELab values of nCDA were all negatively correlated with vigor index and germination rate, where CIELab A^*^ had Pearson’s correlation coefficient of -0.8866 and -0.9340 with vigor index and germination rate, respectively (**Figure 7C**), also revealing the potential of CIELab analysis of nCDA image in the testing of seed vigour.

**Figure 8:**
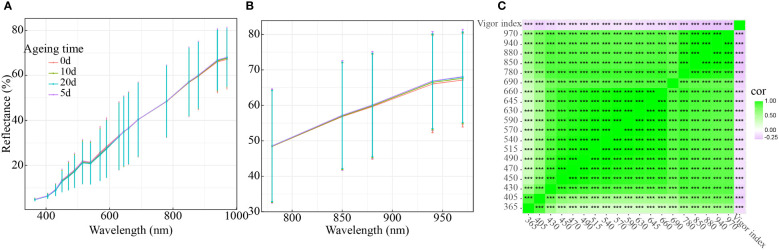
Mean reflectance spectra (from 365 nm to 970 nm) of the 6 rice varieties after 0-, 5-, 10- and 20-day accelerated ageing **(A)**, mean reflectance spectra (from 780 nm to 970 nm) **(B)** and correlation between vigor index of individual seed and spectra **(C)** ***: significant correlation with p<0.001..

The spectral reflection results showed that, there was little variation among different types of accelerated ageing in lower wavelength (**Figure 8A**), while the variation became obvious when it came to higher wavelength (from 780 nm to 970 nm, **Figure 8B**). Compared with non-aged seeds, seeds with accelerated ageing had higher reflection intensity (**Figures 8A, B**), which were in accordance with previously reported researches (ElMasry et al., 2019b). Meanwhile, correlation between vigor index and spectra also illustrated their significantly negative correlation, where the Pearson’s correlation coefficient were from -0.2523 to -0.2994 in wavelength from 780 nm to 970 nm, and much higher than those of lower wavelength (Pearson’s correlation coefficient: from -0.1411 to -0.2267) (**Figure 8C**). Therefore, 780 nm - 970 nm may be the feature spectrum for seed vigour. Since 780 nm - 970 nm has close relationship with fat (Song et al., 2015; Sendin et al., 2018), further researches on material composition and gene expression are required.

Four types of metrics were applied in our study for the purpose of evaluating the capability of our modelling in germination prediction, including TP, FP, TN, FN, and based on which, ACC, Precision, TPR (also knwon as recall), FPR, TNR and FNR were also used for further evaluation.


(2) $$\;ACC=\;\frac{TP+TN}{TP+TN+FP+FN}$$

(3) $$\;Precision=\frac{TP}{TP+FP}$$

(4) $$\;TPR=\frac{TP}{TP+FN}$$

(5) $$\;FPR=\frac{FP}{FP+TN}$$

(6) $$\;TNR=\frac{TN}{TN+FP}$$

(7) $$\;FNR=\frac{FN}{FN+TP}$$

Based on the metrics mentioned above, the MsiFormer proposed in our study was evaluated in comparison with several excellent deep learning methods, *e.g.* ResNet (He et al., 2016), DenseNet (Huang et al., 2017) and EfficientNet (Tan and Le, 2019). ResNet (He et al., 2016) is a convolutional neural network and has good performance in image classification and object recognition. ResNet was designed with many residual blocks that used skip connections to improve accuracy by adding considerable depth, alleviating the problems of gradient disappearance and gradient explosion caused by increasing depth in deep neural networks. DenseNet (Huang et al., 2017) is a densely connected convolutional network which connects each layer to other layers in a feed-forward fashion, where the input of each layer comes from the output of all previous layers. DenseNet reduces the number of parameters and enhances the transfer and reuse of features, and performs well in image classification, object detection, and image generation. EfficientNet (Tan and Le, 2019) is a deep learning method and uses the web search technique NAS to increase the resolution, depth and width of the network simultaneously for better results. EfficientNet is also a general architecture for computer vision that could handle multiple vision tasks including image classification. Based on special design (Mentioned in ‘2. Materials and Methods’), MsiFormer can classify multispectral images containing more channels (22 channels), while ResNet, DenseNet and EfficientNet can only classify RGB images with 3 channels.

According to **Table 3**, MsiFormer surpassed other methods in all evaluation metrics, revealing the great efficiency of our deep learning architecture.

**Table 3:** Evaluation of MsiFormer in predictions of seed germination prediction in comparison with other methods.

Method	TP	TN	FP	FN	ACC	Precision	TPR	FPR	TNR	FNR
ResNet50	52	55	5	8	89.17	91.23	86.67	8.33	91.67	13.33
DenseNet121	56	54	6	4	91.67	90.32	93.33	10.00	90.00	6.67
EfficientNetb4	59	32	28	1	75.83	67.82	98.33	46.67	53.33	1.67
MsiFormer	59	54	6	1	94.17	90.77	98.33	10.00	90.00	1.67

**Figure 9:**
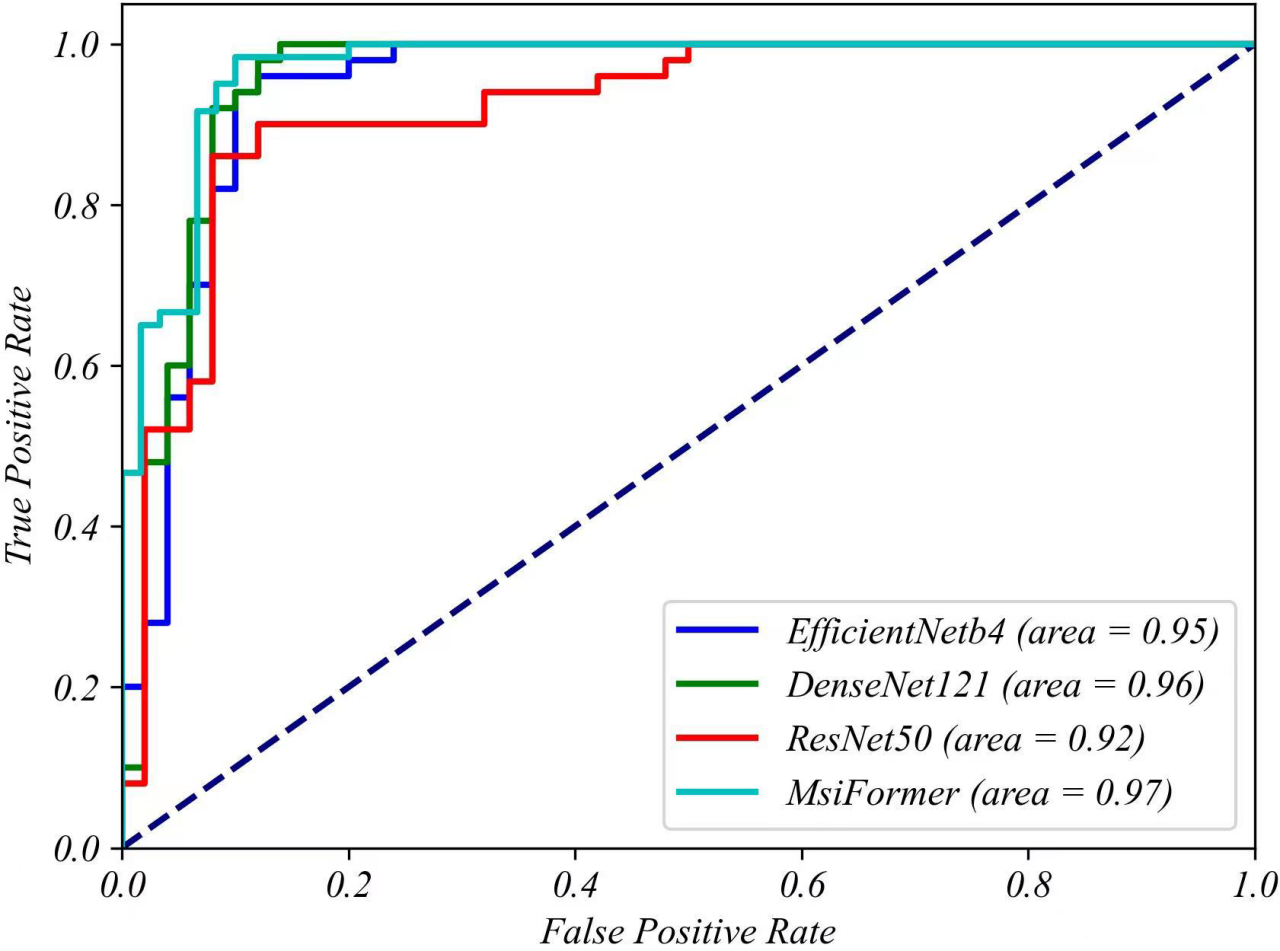
The ROC plots of different methods in seed germination prediction.

As shown in **Figure 9**, the ROC curve comparison of different methods was plotted, where TPR and FPR were taken as ordinate and abscissa, respectively. Generally, the closer the ROC curve is to the top left, the higher the accuracy; the higher the AUC value (AUC: the area under curve, or the area under the ROC curve), the better the prediction ability of the method. Compared with other methods, MsiFormer had a better ROC curve, and higher AUC value as well. Therefore, MsiFormer is robust in predicting the germination of seeds individually, and germination rate of a given variety can be calculated accordingly.

## 4 Discussion

Rice is a very important food crop. In recent years, rice seed vigour raises increasing concerns due to the growing application of hybrid rice and direct sowing, along with environmental deteriorating and arable land decreasing. However, rice seed vigour is mainly tested with the aid of conventional methods, including visual detecting via germination rate and vigor index.

Conventional testing methods require intricate procedures and skilled staff, which are sample-destructive, labor-intensive and time-consuming (Baek et al., 2019; Xia et al., 2019). The importance of developing new technologies for seed vigour determination is recognized by both the International Seed Testing Association (ISTA) and Association of Official Seed Analysts (AOSA) (Boelt et al., 2018). In our study, new testing methods based on computer vision were proposed, including CIELab-mediated image analysis of TTC staining and nCDA, and particularly, MsiFormer-based MSI techniques. Compared with conventional methods, our new methods can reduce human interference and subjectivity, and thus, enhancing the automation and accuracy.

Our MSI technique, with the integration of Yolo v5 and MsiFormer, was developed in our study. Compared with SVM, ANN, *etc*., our MSI technique can improve automation with little pretreatment, including manual threshold setting, extraction of feature spectrum, *etc*. Based on our MSI technique, ROI segmentation can be implemented in high efficiency, and then seeds with no germinating ability can be screened out from the sound ones. Since conventional testing of rice seed vigour is based on single seed and adopt the strategy of small sampling representing the overall batches or bulks of seeds, it is not only tedious and time-consuming, but also neglects variation among individual seeds. According to our results, mean coefficient of variation (CV) of vigor index of individual seed reached 64.87%. Therefore, development of on-site seed screening to detect the variations among seed lots and among individual seeds within a seed lot will be in great favor of improving the accuracy of rice seed vigour testing, and providing convenience for seed production and processing. Besides ROI segmentation, our MsiFormer also offers a new prediction model for germination prediction with good accuracy of 94.17%, which is better than other deep learning methods. Since our model is to predict whether rice seeds will germinate or not and seed ageing is a continuous process, further research on quantitative discrimination in rice seed vigour is expected.

Most MSI technologies were dependent on prediction modelling and multispectral images. In our study, CIELab-mediated image analysis of TTC staining and nCDA were proposed as other options in case that prediction modelling and multispectral images are unavailable.

## 5 Conclusion

Our study further revealed the great demand of automated and accurate methods in rice seed vigour testing, and offered new computer vision-based methods accordingly, which can reduce human interference and subjectivity.

## Data availability statement

The datasets presented in this study can be found in online repositories. The names of the repository/repositories and accession number(s) can be found below: https://github.com/LiaoYun0x0/MisFormer.

## Author contributions

YFL and WW conceived and designed the experiments; JQ, CY, XY, HT performed the experiments and conducted the data analysis; YL established the models; YFL and YL wrote the article; WW revised the article. All authors contributed to the article and approved the submitted version.
